# Pilocarpine in Uræmia

**Published:** 1880-02

**Authors:** 


Pilocarpine in Uræmia.—Four cases are reported by Bogehold where the hypodermic injection of pilocarpine was applied to the treatment of uræmia.

1. Girl aged five years, attacked with a grave form of uræmia following scarlet fever. An injection of one-tenth of a grain of pilocarpine was followed by the stoppage of the convulsions, and the child regained consciousness. Some days after a new attack occurred which was treated the same way with the same results. These injections were employed every day for twenty days, when the convulsions failed to return. Ten days after the cessation of treatment, the albuminuria disappeared and recovery was complete.

2. Boy twelve years old became uræmic during convalescence from scarlet fever. An injection of one-sixth of a grain of pilocarpine caused the stoppage of the convulsions at the end of six minutes, and on two returns was equally successful; the patient ultimately recovering.

3. A seven year old child. Injection of pilocarpine stopped the convulsions but the other symptoms persisted and death followed on coma.

4. A seven year old girl subject to chronic nephritis. Pilocarpine failed to have any influence on the convulsions. Blood-letting was tried also without effect, and the child died.—*Deutsch Med. Wochenschrift*, No. 26, 1879.

